# Ikaros Zinc Finger Transcription Factors: Regulators of Cytokine Signaling Pathways and CD4^+^ T Helper Cell Differentiation

**DOI:** 10.3389/fimmu.2019.01299

**Published:** 2019-06-06

**Authors:** Michael D. Powell, Kaitlin A. Read, Bharath K. Sreekumar, Kenneth J. Oestreich

**Affiliations:** ^1^Fralin Biomedical Research Institute, Virginia Tech Carilion, Roanoke, VA, United States; ^2^Translational Biology, Medicine, and Health Graduate Program, Virginia Tech, Blacksburg, VA, United States; ^3^Biomedical and Veterinary Sciences Graduate Program, Virginia-Maryland College of Veterinary Medicine, Virginia Tech, Blacksburg, VA, United States; ^4^Department of Biomedical Sciences and Pathobiology, Virginia-Maryland College of Veterinary Medicine, Virginia Tech, Blacksburg, VA, United States; ^5^Virginia Tech Carilion School of Medicine, Roanoke, VA, United States

**Keywords:** CD4^+^ T helper cells, differentiation, gene regulation, Ikaros zinc finger (IkZF) transcription factors, cytokines

## Abstract

CD4^+^ T helper cells are capable of differentiating into a number of effector subsets that perform diverse functions during adaptive immune responses. The differentiation of each of these subsets is governed, in large part, by environmental cytokine signals and the subsequent activation of downstream, cell-intrinsic transcription factor networks. Ikaros zinc finger (IkZF) transcription factors are known regulators of immune cell development, including that of CD4^+^ T cell subsets. Over the past decade, members of the IkZF family have also been implicated in the differentiation and function of individual T helper cell subsets, including T helper 1 (T_H_1), T_H_2, T_H_17, T follicular (T_FH_), and T regulatory (T_REG_) cells. Now, an increasing body of literature suggests that the distinct cell-specific cytokine environments responsible for the development of each subset result in differential expression of IkZF factors across T helper populations. Intriguingly, recent studies suggest that IkZF members influence T helper subset differentiation in a feed-forward fashion through the regulation of these same cytokine-signaling pathways. Here, we review the increasingly prominent role for IkZF transcription factors in the differentiation of effector CD4^+^ T helper cell subsets.

## Introduction

The seminal discovery by Mosmann and Coffman that naïve CD4^+^ T cells could differentiate into either T helper 1 (T_H_1) or T_H_2 subsets launched an area of immunological investigation aimed at understanding the mechanisms underlying the functional diversity of CD4^+^ T helper cell populations (1). In the past three decades, the original T_H_1 and T_H_2 dichotomy has been expanded to include additional subsets such as T_H_17, T follicular helper (T_FH_), and regulatory T (T_REG_) cell populations (2–4). The diverse functions performed by these populations permit a highly tailored pathogen-specific immune response to bacterial, viral, and parasitic infections. Conversely, dysregulated T helper cell responses have been implicated in a number of autoimmune disorders, including type 1 diabetes, multiple sclerosis, Crohn's disease, and others (3, 5–7). Thus, due to the importance of these cell populations to human health, extensive efforts have been undertaken to better understand how CD4^+^ T helper cell subset differentiation is regulated.

Generally, it is recognized that the differentiation of effector CD4^+^ T cell populations requires three signals. Two of these signals are derived from direct cell-to-cell contact with an antigen-presenting cell (APC), in the form of T cell receptor and co-stimulatory receptor activation (8, 9). Importantly, the third signal, derived from the cytokine environment, drives CD4^+^ T helper cell subset specification through the activation of cytokine-specific transcription factor networks. Association of cytokines with their specific receptors results in the activation of Janus kinase/Signal Transducer Activator of Transcription (JAK/STAT) pathways, in which JAKs phosphorylate members of the STAT factor family (10). This ultimately leads to dimerization and translocation of STAT factors into the nucleus, where they activate the expression of subset-specific genes including those encoding “lineage-defining” transcription factors, which are required for the differentiation of each T helper cell subset (11, 12).

As with STAT transcription factors, members of the Ikaros Zinc Finger (IkZF) transcription factor family have well-documented roles in the development of immune cell populations (13–15). Ikaros, the founding member of the family, was initially shown to be required for lymphoid cell development, as mice expressing a dominant negative form of Ikaros failed to produce early T and B lymphocyte progenitors, as well as Natural Killer cells (16, 17). In the following decades, four proteins with a high degree of homology to Ikaros were identified and now comprise the IkZF family of transcription factors: Ikaros (encoded by the gene *Ikzf1*), Helios (*Ikzf2*), Aiolos (*Ikzf3*), Eos (*Ikzf4*), and Pegasus (*Ikzf5*) (18–22).

**Figure 1:**
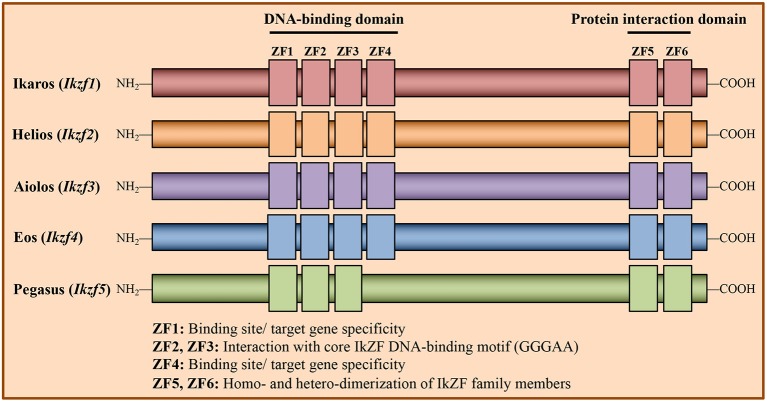
Structure of Ikaros Zinc Finger transcription factor family members. Members of the Ikaros Zinc Finger (IkZF) family of transcription factors are known regulators of hematopoietic cell development, including that of CD4^+^ T cells. The IkZF family consists of five members: Ikaros (encoded by the gene *Ikzf1*), Helios (*Ikzf2*), Aiolos (*Ikzf3*), Eos (*Ikzf4*), and Pegasus (*Ikzf5*). These factors contain N-terminal zinc finger (ZF) domains, which are responsible for mediating direct interactions with DNA, and C-terminal ZFs, which facilitate homo- and heterodimerization between IkZF family members. Of the N-terminal zinc fingers, ZF2 and ZF3 mediate direct interaction with the core IkZF DNA binding motif (GGGAA), while ZF1 and ZF4 regulate factor binding site/target gene specificity.

Structurally, IkZF family members contain both an N-terminal zinc finger (ZF) DNA-binding domain and a C-terminal ZF protein-protein interaction domain (Figure 1) (23). This distinct structure confers diverse functional capabilities, as IkZF family members can both positively and negatively regulate gene expression through direct interactions with DNA, as well as by forming transcriptional complexes with other proteins. Mechanistically, IkZF factors have been shown to regulate gene expression by (i) remodeling chromatin structure through association with chromatin remodeling complexes such as the nucleosome remodeling deacetylase (NuRD), (ii) interacting with and promoting the activity of the RNA Pol II transcription initiation complex, and (iii) inducing chromosome conformational changes by mediating interactions between distal *cis*-regulatory regions (14, 15, 24–26).

**Figure 2:**
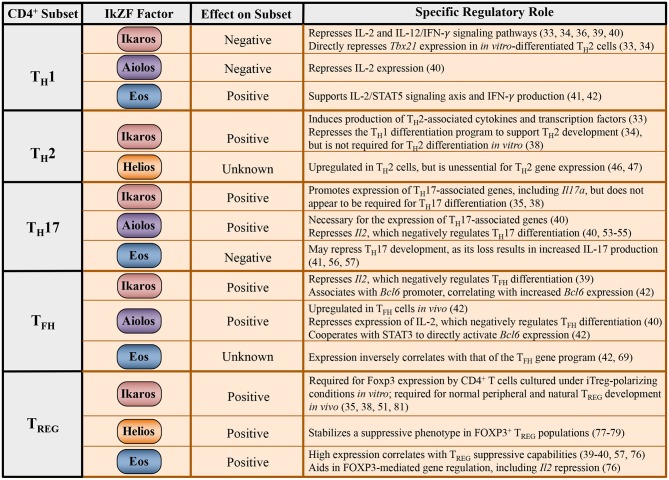
Regulation of CD4^+^ T cell differentiation programs by IkZF family members. Tabulated summary of the known effects of IkZF family members on expression of the T_H_1, T_H_2, T_H_17, T_FH_, and T_REG_ gene programs, including associated cytokines and transcription factors. The broad impact of each IkZF factor on the differentiation program of individual T helper cell subsets is also provided, if known.

Recent research efforts have examined potential roles for IkZF family members in regulating the development of effector CD4^+^ T cell populations (Figure 2). Intriguingly, many of these studies point to mechanisms whereby IkZF factors propagate T helper cell subset differentiation via the modulation of cytokine signaling pathways. Here, we review the literature describing roles for IkZF members in the regulation of CD4^+^ T cell differentiation, with an emphasis on the interplay that exists between IkZF transcription factors and cell-specific cytokine signaling pathways.

## T Helper 1 (T_H_1) Cells

The differentiation and function of T_H_1 cells is critical for effective adaptive immune responses against intracellular pathogens, including viruses, bacteria, and parasites. T_H_1 differentiation is dependent upon extracellular signals from the cytokine IL-12, which lead to activation of the transcription factor STAT4 (27–29). Upon activation, STAT4 dimerizes and translocates to the nucleus where it directly activates expression of *Tbx21*, the gene encoding the T_H_1 lineage-defining transcription factor T-bet. T-bet directly induces the expression of the key T_H_1 effector cytokine Interferon-gamma (IFN-γ), which functions in a feed-forward fashion, as autocrine IFN-γ signals lead to STAT1 activation and further T-bet expression (30). The production of IFN-γ allows effector T_H_1 cells to initiate anti-intracellular pathogen responses including increased activation and proliferation of macrophages, as well as the activation of CD8^+^ T cell populations, which are responsible for the elimination of infected cells. In addition to IL-12 signaling, T_H_1 cell differentiation is dependent upon autocrine signals from IL-2, the expression of which is induced upon T cell receptor/co-receptor activation. IL-2 signaling results in the activation of STAT5, which, like STAT4, induces expression of genes encoding both transcription factors (including Blimp-1) and cytokine receptors (including components of the IL-12 and IL-2 receptor complexes) that are required for continued commitment to the T_H_1 lineage (31, 32).

### IkZF Factors in T_H_1 Cell Differentiation and Function

Of the IkZF family members, Ikaros has been most extensively linked to aspects of T_H_1 cell development. It has been shown in a number of experimental settings that loss of Ikaros expression and/or function results in increased T-bet expression, suggesting that Ikaros plays a role in negatively regulating T_H_1 differentiation (33–35). Specifically, overexpression of a dominant negative form of Ikaros in T_H_2 cells resulted in increased T-bet expression, while Ikaros^null^ *in vitro*-differentiated T_H_2 cells expressed increased T-bet as compared to wildtype controls. Supporting a role for Ikaros in the direct regulation of T-bet expression, Ikaros has been shown to directly bind to the *Tbx21* promoter in *in vitro*-polarized T_H_2 cells (33, 34). However, Ikaros was noticeably absent from the *Tbx21* promoter in *in vitro* differentiated T_H_1 cells, for which T-bet expression is required (34). Mechanistically, the association of Ikaros with the *Tbx21* promoter may be related to alterations in chromatin structure, as another study found increased enrichment of the repressive chromatin mark H3K27^me3^ at this locus upon Ikaros binding in thymocyte populations (36). However, whether this mechanism is conserved in CD4^+^ T cell populations is unclear. Regardless, the collective data support a role for Ikaros in the negative regulation of T_H_1 cell differentiation through direct repression of T-bet expression.

In addition to regulating T_H_1 differentiation pathways, Ikaros has been shown to negatively regulate expression of the T_H_1 effector cytokine, IFN-γ. Ikaros enrichment was observed at predicted *Ifng* regulatory regions in T_H_2 cells, and the *Ifng* promoter displayed reduced methylation in T_H_2 cells expressing a dominant negative form of Ikaros (33, 34). Furthermore, Ikaros^null^ T_H_2 cells were shown to exhibit increased IFN-γ production, as well as an increase in both T-bet and STAT1 transcript expression as compared to WT controls (33, 34). In further support of a T-bet-independent role for Ikaros in regulating *Ifng* expression, it has been shown that overexpression of wildtype Ikaros in Ikaros^null^ T_H_2 cells results in reduced IFN-γ production in the absence of a significant impact on T-bet expression (37). Collectively, these data further support a repressive role for Ikaros in both T_H_1 cell differentiation and function.

It is important to note, however, that all of the above studies utilized germline mutant models to assess the role of Ikaros in regulating T helper cell differentiation programs. Providing further clarity regarding the role of Ikaros in T helper cell differentiation decisions, a recent study assessed the effects of conditional Ikaros knockout exclusively in mature T cell populations on CD4^+^ T cell differentiation and function (38). Curiously, Ikaros-deficient mature T helper cells exposed to T_H_1-polarizing conditions did not exhibit increased T-bet or IFN-γ expression as compared to WT. However, Ikaros-deficient T_H_2 cells displayed increased IFN-γ expression, possibly supporting a role for Ikaros in negatively regulating T_H_1 gene expression in alternative T helper cell subsets, consistent with previous findings (38).

**Figure 3:**
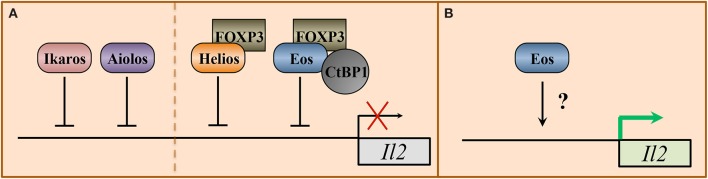
Transcriptional regulation of the interleukin-2 locus by IkZF transcription factors. Signals from the pro-inflammatory cytokine interleukin-2 (IL-2) differentially regulate the expression of T helper cell programs. IL-2 signaling supports the differentiation of T_H_1, T_H_2, and T_REG_ cell subsets, but represses the differentiation of T_H_17 and T_FH_ populations. The Ikaros zinc finger family members Ikaros, Helios, Aiolos, and Eos have all been implicated in regulating IL-2 expression. **(A)** In anergic CD4^+^ and T_H_17 cells, respectively, Ikaros and Aiolos have been shown to directly associate with the *Il2* promoter to repress IL-2 expression. For Ikaros, this association correlates with reduced H3 and H4 acetylation. Aiolos association has been linked to a decrease in both acetylation and the positive histone mark H3K4^me3^ at the *Il2* promoter and a concurrent increase in H3K9^me3^, which is indicative of a transcriptionally inactive locus. In regulatory T (T_REG_) cells, Eos and Helios have both been implicated in IL-2 repression. Mechanistically, Eos forms a protein complex with the T_REG_ lineage-defining transcription factor FOXP3 and C-terminal binding protein (CtBP) to repress *Il2*. As with Aiolos, this repression is associated with reduced H3 and H4 acetylation, reduced H3K4^me3^, and increased H3K9^me3^ enrichment at the *Il2* promoter. Similarly, Helios and FOXP3 are co-enriched at the *Il2* locus in T_REG_ cells and correlate with reduced H3 acetylation, consistent with gene repression. **(B)** In conventional T helper cell populations, in the absence of FOXP3, Eos has been positively linked to the expression of IL-2. However, the mechanism by which Eos influences IL-2 expression is currently unclear.

Illustrating an expanded role for Ikaros in regulating T_H_1 cytokine signaling pathways, Ikaros has also been shown to directly associate with the *Il2* promoter and repress its expression (Figure 3) (39). Loss of Ikaros function was found to result in increased acetylation at the *Il2* promoter, which correlated with increased IL-2 production in anergic T helper cells undergoing TCR stimulation. Similarly, Aiolos has also been shown to directly repress IL-2 expression (40). Given the importance of the IL-2/STAT5 pathway to T_H_1 cell differentiation, these data suggest that Ikaros and Aiolos may also negatively regulate T_H_1 differentiation by repressing autocrine IL-2 signaling.

Unlike Ikaros and Aiolos, the available literature suggests that Eos may function as a positive regulator of the T_H_1 gene program. Specifically, mixed bone marrow chimera studies utilizing a murine experimental autoimmune encephalomyelitis (EAE) model demonstrated that there was a reduction in IFN-γ production by Eos-deficient vs. WT CD4^+^ T cells. Curiously, CD4^+^ T cells in Eos^−/−^ animals displayed no difference in IFN-γ production as compared to those in WT animals during EAE, suggesting that there may be a compensatory mechanism at play in Eos-deficient animals (41). An additional study found that Eos expression correlated with that of T_H_1 genes at both the transcript and protein level (42). Furthermore, Eos was shown to regulate the expression of both IL-2 and CD25 expression in conventional CD4^+^ T (T_CONV_) cell populations (41). Thus, Eos may positively influence T_H_1 gene expression patterns, at least in part, by promoting the IL-2/STAT5 signaling axis. Additional work will be required to determine the precise role of Eos in promoting T_H_1 cell differentiation. Collectively, these studies support opposing roles for Ikaros and Aiolos vs. Eos in regulating T_H_1 cell differentiation and function.

### T Helper 2 (T_H_2) Cells

T_H_2 responses both provide protection against parasitic infection, such as with helminthic worms, and also assist with tissue repair mechanisms following parasite- and inflammation-induced damage (43). T_H_2 differentiation is dependent upon both paracrine and autocrine IL-4 signaling, which results in the activation of STAT6. Once activated, STAT6 directly induces the expression of the T_H_2 lineage-defining transcription factor GATA3 (44). GATA3 then directly activates expression of the T_H_2 effector cytokines IL-4, IL-5, and IL-13, which recruit and activate additional immune cell types, such as macrophages and eosinophils, and promote IgE production to facilitate parasite clearance. As with T_H_1 cells, T_H_2 cell differentiation is also positively regulated by autocrine IL-2/STAT5 signaling, which, in the T_H_2 context, functions to induce GATA3-indepdendent expression of IL-4, as well as the feed-forward induction of IL-4Rα (45).

#### IkZF Factors in T_H_2 Cell Differentiation and Function

Ikaros has been implicated in positively regulating IL-4 production in T_H_2 cells. Specifically, naïve CD4^+^ T helper cells isolated from Ikaros^null^ mice exposed to T_H_2-polarizing conditions were found to exhibit reduced production of IL-4, and direct association of Ikaros with the *Il4* locus was accompanied by increased H3 acetylation (33). Unsurprisingly, this correlated with decreased expression of the T_H_2 transcription factors GATA3 and cMaf (33). As mentioned previously, Ikaros has also been shown to promote T_H_2 lineage specification through direct repression of the T_H_1 lineage-defining transcription factor, T-bet (34). In support of this, in an *in vivo* experimental setting, mice heterozygous for a dominant negative form of Ikaros displayed an inappropriate T_H_1 response to *Shistosoma mansoni* parasitic infection (34). It is important to note that given heterodimeric interactions between IkZF family members, the dominant negative Ikaros protein, which lacks a functional N-terminal DNA binding domain, may disrupt the function of other IkZF factors in this experimental setting. Thus, the precise role of Ikaros in regulating the phenotypes observed in this study is unclear. Additionally, contrary to the above findings, a recent study utilizing a CD4^+^ specific conditional knockout of Ikaros exclusively in mature T cell populations found that Ikaros-deficient T_H_2 cells do not display a defect in either Gata3 or IL-4 expression, although they do produce more IFN-γ, as described above (38). Thus, it is possible that Ikaros deficiency during T cell development, vs. the naïve to effector transition, results in an altered phenotype that makes analysis of individual effector T cell populations difficult. Ultimately, these data suggest that Ikaros may support non-T_H_1 subsets by repressing the Th1 gene program during differentiation.

Similar to Ikaros, Helios expression is upregulated in T_H_2 cells generated in response to ovalbumin immunization *in vivo*, coinciding with the expression of GATA3, cMaf, and IL-4 (46). However, unlike Ikaros, Helios does not seem to be required for induction of the T_H_2 phenotype, as the same study showed that loss of Helios expression had no effect on the expression of T_H_2-associated cytokines and transcription factors at the transcript level (46). Additionally, no difference in IL-4 production was observed between T_H_2-polarized WT and Helios-deficient T cells, further establishing Helios as non-essential for T_H_2 development (47). Thus, although Helios is highly expressed in T_H_2 cells, there is a lack of evidence to support Helios as a regulator of T_H_2 development.

In addition to the production of IL-4, T_H_2 cells mediate their effector functions via secretion of the pro-inflammatory cytokines IL-5 and IL-13 (43). As with the IL-4 studies described above, Ikaros appears to positively mediate T_H_2-associated functions as exposure of naïve CD4^+^ T helper cells isolated from Ikaros^null^ mice to T_H_2 polarizing conditions resulted in reduced production of IL-5 and IL-13 compared to cells from wild-type mice (33). This is perhaps unsurprising, given the requirement for Ikaros for expression of GATA3, which directly regulates the expression of IL-5 and IL-13. Collectively, the above studies suggest that Ikaros positively regulates T_H_2 differentiation and function, both via the activation of IL-4 expression and through repression of the opposing T_H_1 differentiation program.

Finally, as mentioned above, Ikaros has also been implicated in the direct repression of *Il2*, which is critical for T_H_2 differentiation, in activated CD4^+^ T cell populations (39). This is somewhat at odds with the positive role for Ikaros in regulating T_H_2 development, and suggests that Ikaros may serve differential roles across T helper cell subsets.

## T Helper 17 (T_H_17) Cells

T_H_17 cells are essential mediators of immunity at mucosal surfaces, and function to eliminate pathogenic extracellular bacteria and fungi (7, 48). A number of different cytokines have been implicated in T_H_17 cell differentiation, including TGF-β, and IL-6, among others. Signals from these cytokines result in the upregulation of T_H_17 transcriptional network that includes activation of STAT3, which directly regulates a number of genes required for T_H_17 differentiation including the T_H_17 lineage defining transcription factor RORγt (49, 50). Both STAT3 and RORγt are required for the production of the pro-inflammatory T_H_17 effector cytokines IL-17 and IL-22, which recruit and activate immune cells including neutrophils during the course of infection (2).

### IkZF Factors in T_H_17 Cell Differentiation and Function

Recent work suggests that Ikaros is an important regulator of the T_H_17 gene program (35). Specifically, loss of Ikaros expression in *in vitro*-generated T_H_17 cells has been found to result in decreased expression of T_H_17 genes including those encoding RORγt and IL-17. Consistent with the known role for Ikaros in modulating the epigenetic landscape, expression of Ikaros in T_H_17 cells has been shown to correlate with increased enrichment of permissive covalent histone modifications at these loci (35). More recent reports have described a somewhat conflicting role for Ikaros in T_H_17 function (38, 51). In one study, inhibiting the DNA-binding capability of Ikaros had negligible effects on IL-17 production, while the production of IL-22 was increased (51). One possible explanation for the discrepancy presented by this study is the use of mice expressing a mutant Ikaros protein lacking a functional N-terminal ZF4 domain (Ikzf1^Δ*f*4/Δ*f*4^), as opposed to cells from *Ikzf1*^Null^ mice. Zinc fingers 1 and 4 of the N-terminal zinc finger domain of Ikaros are important for binding to specific target genes, while zinc fingers 2 and 3 bind the core consensus sequence GGGAA (14, 15, 52) (Figure 1). Thus, the mutant utilized in the study may have retained some functionality regarding its DNA binding capability, which could explain the lack of an effect on IL-17 production in this setting. Furthermore, when Ikaros was conditionally knocked out in mature T cell populations, the expression of neither *Il17* nor *Rorc* was impacted when Ikaros-deficient CD4^+^ T cells were exposed to T_H_17 polarizing conditions. Curiously, these cells exhibited increased expression of a pathogenic T_H_17 phenotype, including higher levels of T-bet expression and increased IFN-γ production. These data once again support a role for Ikaros in repressing T_H_1 gene expression during T helper cell differentiation (38).

Similar to Ikaros, recent work has established that Aiolos is necessary for the expression of T_H_17-associated genes including those that encode both IL-17a and IL-17f (40). Mechanistically, this study also determined that Aiolos aided T_H_17 lineage commitment, at least in part, through the direct silencing of the *Il2* locus, as IL-2/STAT5 signaling negatively regulates T_H_17 development (40, 53–55). Thus, both Ikaros and Aiolos appear to regulate T_H_17 differentiation through modulation of T_H_17 gene expression and via repression of alternative gene programs.

In contrast to Ikaros and Aiolos, there is evidence to suggest that Eos may negatively regulate T_H_17 differentiation. Specifically, inhibition of Eos expression by the miRNA miR-17 was shown to enhance T_H_17 cell development (56). Furthermore, Eos-deficient T_REG_ populations were found to gain the ability to produce IL-17 (57). Consistent with this finding, Eos-deficient mice were also found to develop more severe EAE, which correlated with an increased presence of IL-17-producing cells in the central nervous system (CNS) (41).

Taken together, the above findings suggest that Ikaros and Aiolos are positive regulators of T_H_17 differentiation and function, while Eos appears to functionally antagonize the development of T_H_17 cell populations.

## T Follicular Helper (T_FH_) Cells

T_FH_ cells play critical roles in the generation of humoral immunity through their specialized ability to provide help to antibody-producing B cells. T_FH_ cells engage in cognate interactions with B cells and produce the cytokine IL-21 to support the formation of germinal centers in secondary lymphoid tissues and B cell production of high-affinity, pathogen-specific antibodies (5, 58). As with T_H_17 development, T_FH_ cell differentiation can be driven by a number of cytokine signals, with two of the more prominent being IL-6 and IL-21 (59–61). Signals received from these cytokines result in subsequent activation of STAT3, which activates the expression of T_FH_ genes including *Bcl6*, which encodes the lineage-defining transcription factor for the T_FH_ cell subset (62–65).

### IkZF Factors in T_FH_ Cell Differentiation and Function

IL-2 signaling is a potent inhibitor of T_FH_ cell differentiation (32, 66–68). Thus, it is not surprising that as antagonists of IL-2 signaling (39, 40), Ikaros and Aiolos have been implicated as positive regulators of T_FH_ cell differentiation. Surprisingly, however, recent work suggests that Aiolos- and Ikaros-mediated regulation of T_FH_ gene expression patterns may occur independently of this IL-2 dependent repressive mechanism. It was shown that the expression of Aiolos and Ikaros correlates with that of the T_FH_ lineage-defining factor Bcl-6, and that both Aiolos and Ikaros directly associate with the *Bcl6* promoter. (42). Furthermore, their enrichment patterns overlapped with those of histone marks consistent with increased chromatin accessibility and gene activation. Intriguingly, it was also shown that Aiolos interacted with STAT3 to form a novel transcriptional complex capable of inducing Bcl-6 expression in CD4^+^ T cell populations (42). Importantly, this study also found that Aiolos expression was increased in T_FH_ cells generated *in vivo* in response to both *Listeria monocytogenes* and influenza infection, further supporting a role for Aiolos in regulating the T_FH_ differentiation program (42).

As observed in other T helper cell populations, Eos appears to oppose the functions of Ikaros and Aiolos in T_FH_ cells as well. Indeed, unlike Ikaros and Aiolos, Eos expression inversely correlates with that of the T_FH_ gene program (42, 69). A recent study utilizing Nr4a-deficient T cells, which display reduced Eos expression, more readily upregulate both T_FH_ gene expression patterns and obtain the ability to support germinal center reactions (69). It is important to note that these cells also exhibit reduced expression of a number of genes in addition to Eos upon Nr4a loss, and thus this phenotype cannot be directly attributed to loss of Eos expression. An additional study demonstrated that Eos-deficient CD4^+^ T cells are less effective producers of IL-2 as compared to their wild-type counterparts. As IL-2 negatively regulates T_FH_ cell differentiation, these findings suggest that Eos may inhibit T_FH_ differentiation via an IL-2-mediated mechanism (Figure 3) (41). Thus, further work is necessary to determine whether Eos functions to directly repress the T_FH_ gene program or, rather, promotes the expression of alternative effector cell phenotypes.

## Regulatory T (T_REG_) Cells

Unlike the pro-inflammatory effector functions of other T helper cell subsets, the primary role of regulatory T cells is to maintain immune tolerance through a number of suppressive mechanisms, including the secretion of anti-inflammatory cytokines such as IL-10 (70, 71). T_REG_ development is driven by signals propagated through TGF-β engagement with its receptor and the resulting expression of T_REG_ specific transcription factors, including the T_REG_ lineage defining transcription factor Forkhead box P3 (FOXP3), and the IL-2 receptor α chain (CD25) (72–74). This stable expression of CD25, in conjunction with their inability to produce IL-2, allows T_REGS_ to act as “IL-2 sinks,” to restrain pro-inflammatory immune responses. While a number of T_REG_ cell subsets have been identified, much recent work has focused on two major subsets: those that arise from the thymus (tT_REGS_) and those that are generated in the periphery (pT_REGS_) (75).

### IkZF Factors in T_REG_ Differentiation and Function

Gene expression analysis via microarray studies revealed that Eos is highly expressed in T_REG_ populations and that it functions as a key component of the FOXP3-mediated gene repression complex (76). Mechanistically, it was shown that Eos forms a protein complex with FOXP3 and C-terminal binding protein (CtBP) to promote gene silencing in T_REG_ cells (76). These findings are consistent with another study demonstrating that Eos functions as a co-repressor in cooperation with Foxp3 to maintain the T_REG_ phenotype and suppressive capabilities. Specifically, it was established that Eos downregulation occurs in T_REG_ cells in response to inflammation, permitting their transition to a Foxp3-expressing T helper-like cell phenotype (57). Furthermore, another study found that knockdown of Eos expression resulted in decreased T_REG_ function and a subsequent accentuation of colitis in mice (76). Intriguingly, a conflicting study found that Eos-deficient mice did *not* exhibit defective T_REG_ development or function, suggesting that another IkZF factor (or factors) may provide a certain level of redundancy (41).

Interestingly, one of the genes targeted by the Eos/FOXP3-repressive complex is the *Il2* locus, which appears to be directly regulated by a number of IkZF factors across T helper cell populations (Figure 3) (39, 40, 76). Furthermore, Eos-mediated repression of IL-2 seems to be dependent on the expression and activity of FOXP3, as Eos has also been shown to positively regulate IL-2 production in FOXP3^−^ conventional T helper cell populations, as discussed previously (Figure 3) (41). Similar to Eos, Helios has also been implicated in repressing *Il2* expression in T_REG_ populations, supporting the possibility of redundant functions between these factors (Figure 3) (77).

Beyond its role in regulating IL-2 production in T_REG_ cells, several studies have revealed that Helios is also required for the stability of a suppressive phenotype in FOXP3^+^ T_REG_ populations (77). Consistent with these findings, the expression of pro-inflammatory cytokines, including IFN-γ, TNF-α, and IL-17 by T_REG_ populations is significantly increased in the absence of Helios, and Helios-deficient mice display increased numbers of activated T cells and germinal center B cells, as well as increased production of autoantibodies (78). Curiously, Helios-deficient T_REG_ cells also exhibit reduced STAT5 activation and Foxp3 expression, the latter of which can be rescued upon overexpression of a constitutively active form of STAT5 (78). Furthermore, the production of IL-17 is significantly higher in human FOXP3^+^Helios^−^ memory T_REGS_ as compared to FOXP3^+^Helios^+^ populations, supporting a role for Helios in negatively regulating IL-17 production in memory T_REG_ populations (79). However, it is important to note that some studies suggest that Helios expression does not always negatively correlate with inflammatory cytokine expression in T_REG_ populations. Indeed, Helios expression is consistent between conventional FOXP3^+^ T_REG_ cells and IL-17-producing T_REG_ cells that co-express FOXP3^+^ and the T_H_17 lineage-defining transcription factor RORγt (FOXP3^+^RORγt^+^ T_REG_), indicating that IL-17 production may be unrelated to Helios expression in this population (80). Collectively, while many findings support a role for Helios in promoting T_REG_ suppressive function by repressing effector cytokine production, further work is necessary to establish how Helios functions across diverse T_REG_ subtypes.

A number of studies have implicated Ikaros in the regulation of T_REG_ cell differentiation. To this end, it has been shown that under iT_REG_-polarizing conditions, Ikaros-deficient CD4^+^ T cells are unable to upregulate Foxp3 expression (35, 38, 81). Importantly, these studies include cells in which Ikaros had been deleted in the germline (35, 81) and also exclusively in mature T cell populations (38). Additionally, one group observed increased enrichment of the repressive chromatin mark H3K27^me2^ at the *Foxp3* promoter in Ikaros^−/−^ naïve CD4^+^ T cells as compared to WT (35). In further support of a positive role for Ikaros in T_REG_ differentiation, it has been shown that Ikaros-deficient animals exhibit reduced numbers of peripheral and natural T_REG_ populations as compared to WT controls (81). Another study found that CD4^+^ T cells expressing a mutant form of Ikaros lacking the N-terminal DNA-binding ZF4 (IkΔZF4) are unable to normally differentiate into iT_REG_ populations (51). Curiously, while mice expressing the IkΔZF4 mutant were found to exhibit increased numbers of total Foxp3^+^ T_REGS_ *in vivo* under steady-state conditions, the number of pT_REGS_ was reduced (51). Thus, the authors suggest that Ikaros may differentially regulate different T_REG_ cell compartments. It is important to note that this study found that CD4^+^ T cells expressing the IkΔZF4 mutant produce significantly higher amounts of IL-21 than their WT counterparts, which negatively regulates iT_REG_ differentiation (51). However, Ikaros-deficient cells were not found to upregulate IL-21 production, indicating that the mechanism underlying the iT_REG_-deficient phenotypes differs between these two studies (51, 81). Indeed, the authors of the first study found that CD4^+^ T cells isolated from the spleens of Ikaros-deficient mice exhibit a reduction in the expression of Foxo1, a transcription factor required for the generation of regulatory T cells (81). The different mechanisms observed between these studies may be attributed to use of the IkΔZF4 mutant, which is known to function as a dominant negative isoform. As discussed in previous sections, this may alter the function of other IkZF family members with which Ikaros interacts and may confound interpretation of the data in this system. Collectively, however, the available data support a role for Ikaros in positively regulating iT_REG_ differentiation.

## Stat/IkZF Factor Regulatory Modules as Candidate Drivers of T Helper Cell Differentiation

**Figure 4:**
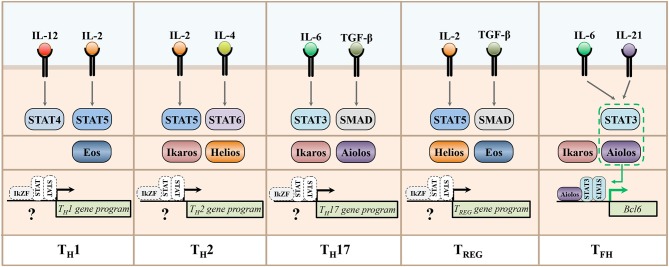
STAT and IkZF regulatory networks underlying the expression of T helper cell gene programs. This schematic depicts representative cytokines implicated in the development of murine T helper cell populations, their downstream STAT signaling pathways, and the Ikaros zinc finger transcription factors recently associated with the differentiation and functions of each subset. A recent study demonstrated that the IkZF factor Aiolos cooperates with STAT3 to induce the expression of *Bcl6*, the lineage-defining transcription factor for the T_FH_ cell type. The differential expression and/or activity of IkZF and STAT factors across T helper cell subsets is suggestive of the intriguing possibility that additional, cell type-specific STAT/IkZF regulatory modules may regulate T helper cell differentiation decisions across subsets.

An interesting aspect of IkZF factors as regulators of T helper cell differentiation is that individual family members appear to perform diverse functions across T helper cell subsets. While the entirety of the mechanisms that permit such broad functionality of IkZF family members is currently unclear, one manner by which this differential regulation may be accomplished is through IkZF factor interactions with other transcription factors responsible for regulating individual T helper cell gene programs. As discussed throughout this review, STAT factors have long been established as cytokine-dependent regulators of effector CD4^+^ T cell differentiation and function (82, 83). Additionally, these factors are known to engage in cooperative mechanisms with other transcription factors to regulate gene expression patterns (84). Thus, STAT factors are prime candidates for potential regulation of IkZF factor function. Indeed, our laboratory recently found that the IkZF factor Aiolos interacts with STAT3 to form a transcriptional complex that induces the expression of Bcl-6, the lineage defining transcription factor for the T_FH_ cell type (42). An interesting feature of this mechanism is that the IL-2/STAT5 signaling axis both inhibits the expression of Aiolos and the activity of STAT3. However, in the absence of IL-2 signaling, increased Aiolos expression and STAT3 activation results in the formation of an Aiolos/STAT3 transcriptional complex capable of inducing a Bcl-6-dependent T_FH_ differentiation program. As detailed in the previous sections, the cytokine-dependent activation of cell-specific STAT factors is accompanied by alterations to expression and activity of different IkZF family members. Given the fact that members of these transcription factor families are both highly conserved and widely expressed across T helper cell subsets, these findings are suggestive of the intriguing possibility that additional IkZF/STAT factor complexes may broadly regulate the differentiation programs of CD4^+^ T cell populations (Figure 4). Furthermore, IkZF factor expression is not unique to CD4^+^ T helper cells, as these transcription factors are expressed in cells throughout the immune system (13, 14, 85, 86). Thus, the possibility exists that IkZF/STAT transcriptional complexes may represent a conserved regulatory feature that regulates an array of immune responses. Given the prominent role of STAT factor signaling in human disease pathology, understanding the extent to which they cooperate with IkZF factors to regulate immune cell differentiation is an intriguing area for future research (87).

## Concluding Remarks

In the past decade, IkZF family members have emerged as key regulators of CD4^+^ T helper subset development and function. Interestingly, many of these studies have identified IkZF factors as regulators of cytokines, cytokine receptors, and other components of cytokine signaling pathways. Likewise, it is becoming increasingly clear that cytokine signals reciprocally regulate the expression and activities of IkZF transcription factors. These points, coupled with the discovery that IkZF factors can engage in cooperative mechanisms with STAT transcription factors, suggest that IkZF factors may continue to emerge as central players in the regulation of T helper cell differentiation. Further work will be required to determine the extent to which IkZF factors may engage in similar mechanisms to regulate the differentiation and function of cells across the immune system. Importantly, cytokine signaling pathways have been popular targets of immunotherapeutic strategies to treat human diseases ranging from cancer to autoimmunity. Therefore, continued study into the role of IkZF factors in the regulation of immune cell differentiation and function will inform the feasibility of targeting these factors in efforts to promote human health.

## Author Contributions

The manuscript was jointly conceptualized and written by MP, KR, BS, and KO.

### Conflict of Interest Statement

The authors declare that the research was conducted in the absence of any commercial or financial relationships that could be construed as a potential conflict of interest.
